# Characteristics of the Surface Plasmon–Polariton Resonance in a Metal Grating, as a Sensitive Element of Refractive Index Change

**DOI:** 10.3390/ma13081882

**Published:** 2020-04-16

**Authors:** Stefano Bellucci, O. Vernyhor, A. Bendziak, I. Yaremchuk, V. M. Fitio, Y. Bobitski

**Affiliations:** 1 INFN-Laboratori Nazionali Di Frascati, 00044 Frascati, Italy; 2 Department of Photonics, Lviv Polytechnic National University, 79013 Lviv, Ukraine; vernyhor@gmail.com (O.V.); bendziak@polynet.lviv.ua (A.B.); yaremchuk@polynet.lviv.ua (I.Y.); v.m.fitio@gmail.com (V.M.F.); bobitski@polynet.lviv.ua (Y.B.)

**Keywords:** metal grating, resonance, surface plasmon–polariton wave, spectral and angular sensitivities

## Abstract

The resonant excitation of surface plasmon–polariton waves in metal gratings with rectangular and sinusoidal relief was studied. The main characteristics of the resonant excitation of a surface plasmon–polariton wave were obtained using analytical methods due to the fact that the resonance is excited at a grating thickness much smaller than a wavelength (1.064 µm). It is shown that the obtained results are very close to those calculated using numerical methods, e.g., Rigorous Coupled Wave Approach (RCWA). There is a small difference in the numerical data defined by both methods. The difference between the parameters for the two types of gratings is small. New analytical relationships of angular and spectral sensitivities with the change of the refractive index of the medium were obtained, depending on the grating period and the angle of incidence of the light beam. An analytical relationship between the spectral and angular widths of the resonant curves, at full width at half maximum, was determined.

## 1. Introduction

Biochemical reactions occurring in liquid solutions lead to a change in the refractive index, which needs to be measured. For such measurements, a number of devices have been proposed (for example in [1,2]), the operation of which is based on the resonance of surface plasmon–polariton waves [3,4], or on the resonance of waveguide modes, in dielectric gratings on dielectric substrates [5,6,7]. The resonance of surface plasmon–polariton waves is realized in prism structures [3,4,8,9] or in the structure of a dielectric or metal grating on a metal substrate [10]. A thorough review on the various applications of waveguide mode resonance is given in [11]. The characteristics of various optical sensors were analyzed in an extensive review [12]. Sensors operating on the above-mentioned principles are based on the change of the resonant wavelength at a fixed angle of incidence of the test beam, or when the resonant angle is changed, at a fixed wavelength, when the refractive index of the test medium is changing. Moreover, a zero reflection coefficient can be observed at the resonance of surface plasmon–polariton waves with precisely selected structural parameters [13]. The reflection coefficient can be about one at the resonance of waveguide modes in a dielectric grating on a dielectric substrate [14,15]. This can be easily detected by a photosensitive device.

On the basis of classical prism structures [3,4], in which the resonance of surface plasmon–polariton waves is excited, devices for measuring the refractive index of liquids are produced [16]. In order to increase the sensitivity of such sensors, some changes have been proposed. For instance, an additional dielectric layer [8,17] is placed between the metal layer and the test liquid, which may contain holes [17] where the test liquid penetrates. Studies of the prism structure, in which a thin dielectric film with a refractive index smaller than the refractive index of a glass prism is located between the prism and the metal layer, have also been conducted [18]. In such a structure, with the additional dielectric layer, two absorption peaks are observed on the angular dependence of the reflection coefficient at a given wavelength. The reflection coefficients are almost zero under accurately selected parameters of the structure layers. Moreover, the resonant curve is narrower at a smaller angle in the angular dependence of the reflection coefficient than the resonant curve at the larger angle [18]. It is proposed to apply a metal grating on a metal film to increase the sensitivity of the prism structure. The gratings period is a few times smaller compared to the wavelength of the test beam [19]. As a result, there is narrowing of the resonant spectral width. To improve the accuracy measurement, it is proposed to measure the phase change in the occurrence and disappearance of resonance [20,21]. However, these innovations did not significantly raise the sensitivity, compared to the classical prism structure. The sensitivity increases with decreasing refractive index of the prism material in such structures. It is 1.12 rad for aqueous solution at a refractive index of 1.76, and 2.52 rad at a refractive index of 1.479 for fused quartz [6]. In [6], prism structural properties were analyzed for wavelength of 0.6328 µm. The sensitivity was determined by the following expression:

(1) $$S_{\theta }=\frac{\theta_{min}\left(n_{a}+\delta n_{a}\right)-\theta_{min}\left(n_{a}\right)}{\delta n_{a}}$$

where $n_{a}$ is the refractive index of the test medium, and we set to $\delta n_{a}=0.0001$ in the numerical calculations. In the Equation (1), $\theta_{min}$ denotes an angle with the minimal reflection coefficient at the given refractive index of the test liquid $n_{a}$ and given constant wavelength.

Sensors based on the resonance of surface plasmon-polaritons in dielectric or metallic grating on the metal substrate (gold or silver) are characterized by the narrow spectral resonant band [22,23,24,25,26]. It measures about one-nanometer, near the wavelength of 1 µm [27,28]. Sensitivities have been compared for sensors based on the surface plasmon–polariton resonance in both the prism and the grating-based structures [26]. The angular sensitivity of the prism and grating structures is shown to be approximately the same. In [29], the compact and low-cost biosensor based on a novel approach to spectroscopy of surface plasmons was described. This sensor is able to register the changes in the refractive index of 3 × 10^−7^.

The surface plasmon–polariton resonance occurs at small grating thicknesses. For example, the resonant thickness of the metal dielectric grating on the metal substrate is 28.3 nm, with a fill factor of F=0.5 [13]. Apparently, one should expect that the resonant thickness of the metal grating will be even smaller. A grating of this thickness makes a small perturbation in the surface of the metal substrate. Thus, the propagation constant of the surface plasmon–polariton wave, excited in the periodic structure, will be very close to the propagation constant of the flat metal-dielectric boundary. Therefore, many sensor parameters can be determined from a waveguide equation that relates the grating period, the incidence angle of the laser beam on a grating, and the propagation constant of the surface plasmon–polariton wave on the flat metal-dielectric boundary. In such structures, the resonant excitation of the surface plasmon–polariton wave produces significant electric and magnetic fields, which can be of practical use in Raman spectroscopy and in the study of luminescence of a small amount of substance.

Therefore, the purpose of this work is to obtain analytical ratios for determining the sensors parameters. In particular, the sensitivity to the change of the refractive index of the test medium has been defined using the waveguide equation. Moreover, the comparison of the obtained parameters, with the parameters determined using the numerical analysis of light beam diffraction by the periodic structure, was carried out.

## 2. Theoretical Analysis of Plasmon–Polariton Resonance Sensitivity Dependence on the Refractive Index of the Test Medium for a Grating on a Metal Substrate

**Figure 1:**
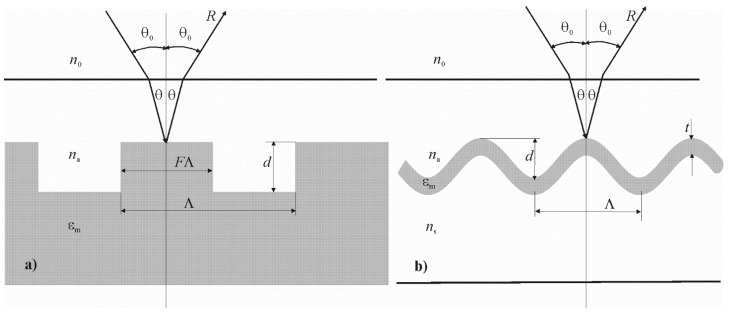
The scheme of periodical structures, where surface plasmon–polariton resonance can be realized: Λ is the grating period, $\varepsilon_{m}$ is the dielectric permittivity of the metal, $n_{a}$ is the refractive index of the test medium, $n_{0}$ is the refractive index of air, θ_0_ is the angle of the beam propagation in air, θ is the angle of the beam propagation in the test medium. Rectangular metal or dielectric grating on a metal substrate, where *F* is the fill factor, *d* is the grating thickness (**a**). Relief grating, where the dielectric-metal boundary changes according to a sinusoidal law, where *d* is the thickness of the metal layer, *A* is the amplitude of the sinusoidal relief, *n*_s_ is the refractive index of the substrate (**b**).

The researched periodical structures, in which the surface plasmon–polariton resonance is excited, are shown in Figure 1.

If the grating period is equal to 1 µm, the grating thickness is 13.4 nm, and the fill factor F is equal to 0.5, then the plasmon–polariton resonance occurs at a wavelength of 1.0109 µm at normal incidence and refractive index $n_{a}=1$ [13]. That is, at the normal beam incidence on the grating, the following expression is true:

(2) $$\frac{2\pi }{\Lambda }≅\frac{2\pi n_{a}}{\lambda }Re\left(\sqrt{\frac{\varepsilon_{m}}{\varepsilon_{m}+\varepsilon_{a}}}\right)$$

where $n_{a}^{2}=\varepsilon_{a},$ λ is the wavelength of the laser beam, $n_{a}$ is the refractive index of the test medium, $\varepsilon_{a}$ is the dielectric permittivity of the test medium, $\varepsilon_{m}$ is the dielectric permittivity of the metal, Λ is the grating period. It should be noted that the difference between the left and right parts of Equation (2) is equal to 0.0062 µm^−1^.

Taking into account that the resonance mostly appears in periodic structures based on silver or gold, and for these metal expressions, $\left|Re\left(\varepsilon_{m}\right)\right|\gg Im\left(\varepsilon_{m}\right)$ and $\left|Re\left(\varepsilon_{m}\right)\right|\gg \varepsilon_{a}$ hold, the following relation is also true:

(3) $$F\left(\lambda \right)=\sqrt{\frac{\varepsilon_{m}\left(\lambda \right)}{\varepsilon_{m}\left(\lambda \right)+\varepsilon_{d}}}≅1$$

where F(λ) is a function of the wavelength, $\varepsilon_{d}$ is the dielectric permittivity of the dielectric medium.

**Figure 2:**
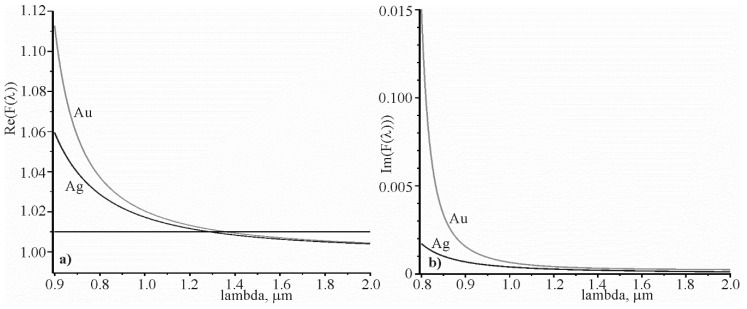
Spectral dependence of $F\left(\lambda \right)=\sqrt{\frac{\varepsilon_{m}\left(\lambda \right)}{\varepsilon_{m}\left(\lambda \right)+n_{a}^{2}}}$ for $n_{a}=1.3242:$ real part of F(λ) (**a**), imaginary part of F(λ) (**b**).

This relation confirms Figure 2, which shows the spectral dependence of Re(F(λ)) and Im(F(λ)) for silver and gold. It can be seen that when λ>1 μm, 1<Re(F(λ))<1.03, and Im(F(λ))<0.001 at $n_{a}=1.3242$.

If the laser beam propagates in a test medium with refractive index $n_{a}$ at incident angle θ, then the waveguide equation can be written as follows:

(4) $$\frac{2\pi n_{a}}{\lambda }sin\;\theta \;\pm \frac{2\pi }{\Lambda }≅\pm \frac{2\pi n_{a}}{\lambda }Re\left(\sqrt{\frac{\varepsilon_{m}}{\varepsilon_{m}+n_{a}^{2}}}\right)$$

Taking into account Equation (3), one can obtain

(5) $$\frac{2\pi n_{a}}{\lambda }sin\theta \;\pm \frac{2\pi }{\Lambda }≅\pm \frac{2\pi n_{a}}{\lambda }.$$

In the case when the laser beam propagates in air with refractive index, $n_{0}≅1$ at angle $\theta_{0}$, and then passes into the test medium, so the following equation will be true:

(6) $$\frac{2\pi n_{0}}{\lambda }sin\;\theta_{0}\;\pm \frac{2\pi }{\Lambda }≅\pm \frac{2\pi n_{a}}{\lambda }Re\left(\sqrt{\frac{\varepsilon_{m}}{\varepsilon_{m}+n_{a}^{2}}}\right)$$

Equation (6) is better to be used, since it is easier to measure the angle of incidence in air. Taking into account Equation (3), we can obtain

(7) $$\frac{2\pi n_{0}}{\lambda }sin\;\theta_{0}\;\pm \frac{2\pi }{\Lambda }≅\pm \frac{2\pi n_{a}}{\lambda }$$

On the basis of Equations (5) and (7), one can calculate the angular sensitivity or spectral sensitivity. The angular sensitivity is the ratio of the change of the resonant angle to the change of refractive index of the test medium, for a given wavelength. The spectral sensitivity is the ratio of the change of the resonant wavelength to the change of the refractive index of the test medium at a constant incidence angle. Let us find the sensitivities based on Equation (7), since angles are measured in air, in reality.

Consider Equation (7) with the sign “+”. The following equation can be obtained by reducing the formula in Equation (7) by 2π; we obtain

(8) $$\frac{n_{0}}{\lambda }sin\;\theta_{0}+\frac{1}{\Lambda }≅\frac{n_{a}}{\lambda }$$

Differentiating Equation (8) by $\theta_{0}$ and $n_{a}$ with a constant wavelength, we will have $n_{0}coscos\;\theta_{0}d\theta_{0}=dn_{a}.$ On the basis of the last equation, expressions for the sensitivity can be given, as follows:

(9) $$\frac{n_{0}}{\lambda }sin\;\theta_{0}+\frac{1}{\Lambda }≅\frac{n_{a}}{\lambda }$$

Similarly, with the sign “−” in Equation (7), we can write

(10) $$S_{\theta -.}=\frac{d\theta_{0}}{dn_{a}}=-\frac{1}{n_{0}cos\;\theta_{0}\;}$$

It can be seen that the angular sensitivity increases, when the incident angle on the grating $\theta_{0}$ increases

(11) $$-n_{0}sin\;\theta_{0}\frac{d\lambda }{\lambda^{2}}\;≅\pm \frac{dn_{a}}{\lambda }\mp \frac{n_{a}d\lambda }{\lambda^{2}}$$

The spectral sensitivity can be determined on the ground using Equation (8) by differentiating it by $n_{a}$ and λ at constant angle $\theta_{0}$. Sensitivities with signs “+” and “−” will be the same. Equation (11) can be written in a more convenient form, as follows:

$$\left(n_{0}sin\;\theta_{0}\;\mp n_{a}\right)\frac{d\lambda }{\lambda }≅\mp dn_{a}\;$$

Taking into account that $\frac{sinn_{0}sin\;\theta_{0}\;}{\lambda }\mp \frac{n_{a}}{\lambda }=\mp \frac{1}{\Lambda },$ the spectral sensitivity can be found from Equation (11) as follows:

(12) $$S_{\lambda +}=S_{\lambda -}=\frac{d\lambda }{dn_{a}}=\frac{\lambda }{n_{0}sin\;\theta_{0}\;\mp n_{a}}=\Lambda$$

The spectral sensitivity increases with increasing wavelength, as well as with the grating period, as follows from Equation (12).

The relationship between spectral width $\delta \lambda_{0.5}$ of the resonant absorption and angular width $\delta \lambda_{0.5}$ can be determined on the basis of Equation (7). Let us consider differentiating Equation (7) by wavelength and angle, with constant $n_{a}$. As a result, we obtained

(13) $$-\frac{2\pi n_{0}sin\;\theta_{0}\;}{\lambda^{2}}\delta \lambda_{0.5}+\frac{2\pi n_{0}cos\;\theta_{0}\;}{\lambda }\delta {\theta_{0}}_{0.5}≅-\frac{2\pi n_{0}}{\lambda^{2}}\delta \lambda_{0.5}$$

After simple algebraic transformations of Equation (13), we can write

(14) $$\delta \lambda_{0.5}=n_{0}\Lambda cos\;\theta_{0}\delta {\theta_{0}}_{0.5}\;$$

Let us compare $\frac{S_{\lambda }}{\delta \lambda_{0.5}}=\frac{\Lambda }{n_{0}\Lambda cos\;\theta_{0}\delta {\theta_{0}}_{0.5}\;}$ and $\frac{S_{\theta }}{\delta {\theta_{0}}_{0.5}}=\frac{\frac{1}{n_{0}cos\;\theta_{0}\;}}{\delta {\theta_{0}}_{0.5}}$. It can be seen that the following equality is fulfilled:

(15) $$N=\frac{S_{\lambda }}{\delta \lambda_{0.5}}=\frac{S_{\theta }}{\delta {\theta_{0}}_{0.5}}$$

where *N* is the ratio between the sensitivity and the spectral or angular width.

The latter ratio is crucial for the sensors, because it determines the sensor’s suitability for measuring the change in the refractive index of the test medium. The larger the value, the more accurately the change in refractive index can be measured. It is intuitively perceived that Equation (15) is valid for such sensors based on resonance phenomena. However, if $\delta \lambda_{0.5}$ or $\delta {\theta_{0}}_{0.5}$ are too small, it may be difficult to measure such narrow resonances. 

The above-mentioned analytical ratios are applicable to gratings with various relief characteristics, in the case when the grating does not significantly disturb the planarity of the metal substrate. Generally speaking, the difference between the sensitivities of surface plasmon–polariton resonance sensors for different grating types can only be determined by diffraction analysis, such as Rigorous Coupled Wave Analysis (RCWA). It should be noted that the parameters should be close to each other.

For the full characterization of sensors based on surface plasmon–polariton resonance, it is necessary to have a spectral or angular dependence of the reflection coefficient (absorption). The spectral and angular widths of the resonant response can be found on the basis of it. The remaining parameters are determined by Equations (9), (12), (14), and (15) if Equations (4) and (6) are satisfied. In this case the surface plasmon–polariton resonance occurs in the periodic structure.

**Figure 3:**
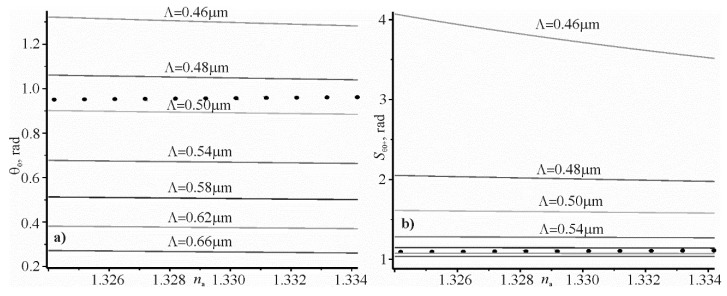
The dependence of the resonance angle at the excitation of the surface plasmon–polariton wave (**a**) and dependence of the sensitivity (**b**) on the refractive index $n_{a}$ of aqueous solution for several grating periods. The black circles reflect the resonance angles and sensitivities of the prism structure. The same color curves correspond to the same grating periods.

The angular dependence on the refractive index of the researched aqueous solution $n_{a}$, calculated by Equation (7) for different grating periods are shown in Figure 3a,b shows the dependence of the sensitivity $S_{\theta 0+}$ on the refractive index $n_{a}$, calculated using Equation (9) for the same grating periods, as shown in Figure 3a. The laser wavelength is 1.064 µm. At this wavelength, the water refractive index is 1.3239 [30] and 1.3245 [31]. Therefore, the refractive index of water is taken as 1.3242 in the calculations. The black dots in Figure 3 correspond to the angular dependences and the sensitivity of the refractive index $n_{a}$, for the prism structure with the refractive index of prism glass BAF10 1.654, and for the silver film thickness of 51 nm. The dielectric constant of silver is equal to −59.3034 + *i*1.280 at a wavelength of 1.064 µm [32,33,34]. At such a thickness of the silver film, the reflection coefficient from the silver film, at the resonant angle, is zero. The width of the resonant curve is 0.00204 rad at full width at half maximum.

It can be seen that the predicted sensitivity of periodic structures in which surface plasmon–polariton waves are excited may be higher than in prism structures. In addition, in such structures the strong electromagnetic fields can be excited by plasmon–polariton resonance. It can be successfully applied in Raman spectroscopy or in luminescence studies.

## 3. Numerical Modeling of Surface Plasmon–Polaritons Resonance in Periodic Structures with Metal

Some results of a numerical study conducted by RCWA on the resonance of the surface plasmon–polariton wave for a silver substrate are reported in [13]. More detailed studies were performed using the finite element method (FEM). The parameters of the structure and the resonant wavelengths are shown in Table 1 for the silver grating.

Comparisons of Columns 5 and 6 show a good match of the resonant wavelengths determined by the two methods. The angle of incidence of the optical wave is normal in all calculations. Therefore, the results of the RCWA and FEM calculations can be trusted, as these are radically different methods and only the “correct results” can match. All the following calculations are made for silver gratings.

**Figure 4:**
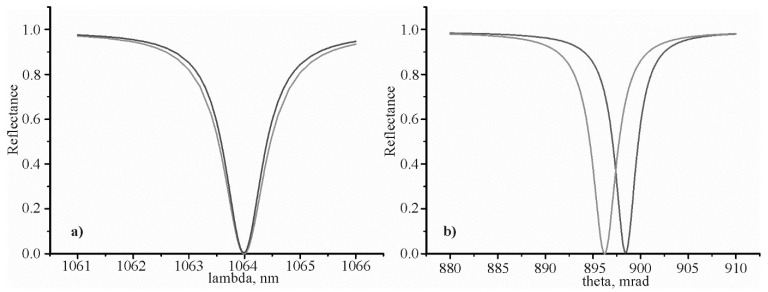
The spectral dependence of the reflection coefficient (**a**) and the angular dependence of the reflection coefficient (**b**). The resonant wavelength is 1.064 µm. The blue color curves correspond to the grating shown in Figure 1a. The red color curves correspond to the grating shown in Figure 1b.

The spectral and angular dependences of the reflection coefficient on the sinusoidal (red color) and rectangular grating profile (blue color) are shown in Figure 4. The reflection coefficient is zero at a resonant wavelength corresponding to a certain resonant angle.

The grating parameters and resonant parameters are shown in Table 2. The spectral resonant curve width $\delta \lambda_{0.5}$ at 0.5 level, given in column 7 of Table 2, was determined by Equation (14) $\delta \lambda_{0.5}=n_{0}\Lambda coscos\;\theta_{0}\delta {\theta_{0}}_{0.5}$.

Analyzing the data in Figure 3 and Table 2, it can be noted that the resonant parameters for both gratings are quite close. It is expected, since the wavelength for silver $\sqrt{\frac{\varepsilon_{m}\left(\lambda \right)}{\varepsilon_{m}\left(\lambda \right)+\varepsilon_{d}}}≅1$ at a wavelength of 1064 µm. A small difference of the expression $\sqrt{\frac{\varepsilon_{m}\left(\lambda \right)}{\varepsilon_{m}\left(\lambda \right)+\varepsilon_{d}}}$ with respect to 1 explains the small difference between the resonant angles 898.4 – 896.2 = 2.2 mrad. It should be expected that the corresponding difference will be even smaller, when the wavelength increases to 2 µm. However, small differences between the parameters of Columns 3–7 are due to the different levels of grating relief. However, the difference between Columns 5 and 7 is approximately equal to 0.01 nm. This confirms the correctness of Equation (14). As it was suggested in the introduction, the resonant thicknesses of metal gratings are close for these types of gratings at *F* = 0.5. They are quite small, i.e., 10 and 12 nm, respectively.

Table 3 shows the sensitivities $S_{\theta },\;S_{\lambda }$ obtained on the basis of Equations (9) and (12), and also due to the numerical analysis. For instance, $S_{\theta }$ was calculated using Equation (1), and the ratios $\frac{S_{\theta }}{\delta \theta_{0}{}_{0.5}}$ and $\frac{S_{\lambda }}{\delta \lambda_{0.5}}$.

In Table 3, the parameters of Columns 2, 3, 4, 6, and 8 were obtained as a result of numerical calculations of the grating diffraction analysis. Columns 5 and 7 were obtained using Equations (12) and (9). The values $\frac{S_{\theta }}{\delta {\theta_{0}}_{0.5}}\;$ and $\frac{S_{\lambda }}{\delta \lambda_{0.5}}$ in the denominator, Columns 9 and 10, respectively, were calculated on the basis of the data of Columns 3, 4, 5, and 7. The numerators of Columns 9 and 10 were calculated on the basis of the data of Columns 3, 4 ,6, and 8. It can be concluded from Table 3 that the difference between Columns 5 and 6 is less than 6 nm. Therefore, the relative difference is larger than 1%. However, the relative difference between Columns 7 and 8 is about 2.7%. The relative difference between the denominators of Columns 9 and 10 is not more than 1.3%, and the difference between the numerators of the same columns is not more than 2.1%. The results of analysis of Rows 4 and 5 of Table 3 for Columns 9 and 10 show a relative difference of less than 14%. Thus, the grating relief still has some influence on the results of the calculations. It should be noted that the grating relief was not taken into account in Equations (2)–(15).

## 4. Conclusions

The comparison of the angular sensitivity of prism and grating structures in which the surface plasmon–polariton resonance is excited indicates that the grating structure has a higher sensitivity, due to the decrease of the grating period in accordance with Figure 3b. Therefore, the grating period allows one to control the sensitivity. The real part of the expression $\sqrt{\frac{\varepsilon_{m}\left(\lambda \right)}{\varepsilon_{m}\left(\lambda \right)+n_{a}^{2}}}$ tends to one for wavelengths larger than 1 µm. The imaginary part of this expression tends to zero with increasing wavelength, as shown in Figure 2. However, it can be assumed that $\sqrt{\frac{\varepsilon_{m}\left(\lambda \right)}{\varepsilon_{m}\left(\lambda \right)+n_{a}^{2}}\;}≅1$ even for a wavelength of 1.064 µm. This assumption allowed us to obtain new analytical expressions that relate the angular sensitivity to the incidence angle of the beam, and the spectral sensitivity to the grating period. Using the approximate ratio $\sqrt{\frac{\varepsilon_{m}\left(\lambda \right)}{\varepsilon_{m}\left(\lambda \right)+n_{a}^{2}}\;}≅1$, we could analytically connect the resonant spectral width and the resonant angular width on the full width at half maximum with the simple equation $\delta \lambda_{0.5}=n_{0}\Lambda coscos\;\theta_{0}\delta {\theta_{0}}_{0.5}\;$, and obtain a fundamental ratio $N=\frac{S_{\lambda }}{\delta \lambda_{0.5}}=\frac{S_{\theta }}{{𝛿\theta_{0}}_{0.5}}.$ This ratio is crucial for the sensors, because it determines the sensor’s suitability for measuring the change in the refractive index of the test medium. It is intuitively understood that this relation can characterize other types of sensors, the work of which is based on resonance. It can be the resonance of surface plasmon polaritons in the prism structure, or the resonance of waveguide modes in the dielectric grating.

The analytical expressions obtained were confirmed by numerical calculations. They were used to determine the angular and spectral sensitivities, as well as the widths of the resonant curves, which are shown in Table 2 (Columns 5 and 7) and Table 3 (Columns 5–10).

It should be noted that the above analytical expressions were obtained from the condition of insignificant disturbance of the metal surface planarity of the grating. This assumption is justified, since the thickness of the gratings is 10 and 12 nm. Such values are much smaller than the wavelength and the grating period. Therefore, the equations obtained give the same parameter values for both types of gratings. However, there is a small difference in the numerical calculations for both types of gratings, as shown in Figure 3. There are more narrow spectral and angular resonant curves for the sinusoidal grating, compared to the grating with rectangular relief. In addition, there is a small difference of 2.2 mrad between resonant angles. This small difference in the numerical and analytical calculations for the two types of gratings can be seen when comparing the data of Rows 3 and 4 of Table 2 and Table 3.

## Figures and Tables

**Table 1:** Parameters of periodic structures for silver substrate according to Figure 1a, and the resonant wavelengths determined by Rigorous Coupled Wave Analysis (RCWA) and finite element method (FEM). The grating period is 1 µm for all examples in Table 1.

No	*d*, nm	$\varepsilon_{a}$	$\varepsilon_{m}$	*F*	*λ*, µm, (RCWA) [13]	Λ, µm (FEM)
	1	2	3	4	5	6
1	50	1	Ag	0.143	1.0035	1.0039
2	13.4	1	Ag	0.5	1.0109	1.0107

**Table 2:** Grating parameters and resonant parameters corresponding to the resonant curves are shown in Figure 3.

Parameter	Λ, nm	*F*	d, nm	$\theta_{0},\;\mathrm{mrad}$	$\delta \lambda_{0.5}$, nm	$\delta {\theta_{0}}_{0.5},\;\mathrm{mrad}$	$\delta \lambda_{0.5},\;\mathrm{nm}\;(\mathrm{Equation}\;(14))$
No	1	2	3	4	5	6	7
Figure 1a	500	0.5	10.0	896.2	0.95	3.07	0.959
Figure 1b	500	0.5	12.0	898.4	0.83	2.7	0.841

**Table 3:** Parameters characterizing the sensitivity of both grating types compared to the change of the refractive index of the test medium.

Parameter	Λ, nm	$\theta_{0},\;\mathrm{mrad}$	$\delta \lambda_{0.5},\;\mathrm{nm}$	$𝜹{\theta_{0}}_{0.5},\mathrm{mrad}$	$S_{\lambda },\;\mathrm{nm}\;(\mathrm{Equation}\;(10))$	$S_{\lambda },\;\mathrm{nm}$	$S_{\theta },\;\mathrm{rad}\;(\mathrm{Equation}\;(7))$	$S_{\theta },\;\mathrm{rad}$	$\frac{S_{\theta }}{\delta \theta_{0}{}_{0.5}}$	$\frac{S_{\lambda }}{\delta \lambda_{0.5}}$
No	1	2	3	4	5	6	7	8	9	10
Figure 1a	500	896.2	0.95	3.07	500	500	1.601	1.65	537/521	526/526
Figure 1b	500	898.4	0.83	2.7	500	505.6	1.605	1.65	611/594	608/602
